# Distinct Phenotypic and Molecular Characteristics of CD34^−^ and CD34^+^ Hematopoietic Stem/Progenitor Cell Subsets in Cord Blood and Bone Marrow Samples: Implications for Clinical Applications

**DOI:** 10.3390/diagnostics15040447

**Published:** 2025-02-12

**Authors:** Ameera Gaafar, Fatheia Nabeil Hamza, Rama Yousif, Zakia Shinwari, Aminah Ghazi Alotaibi, Alia Iqniebi, Khalid Al-Hussein, Amer Al-Mazrou, Pulicat Subramanian Manogaran, Tusneem Elhassan, Marcela Marquez-Méndez, Mahmood Aljurf, Hind Al-Humaidan, Ayodele Alaiya

**Affiliations:** 1 Cell Therapy and Immunobiology Department, King Faisal Specialist Hospital and Research Center, P.O. Box 3354, Riyadh 11211, Saudi Arabia; 2 Biochemistry and Molecular Medicine Department, Alfaisal University, P.O. Box 3354, Riyadh 11211, Saudi Arabia; 3 Cancer Center for Excellence, King Faisal Specialist Hospital and Research Center, Riyadh 11211, Saudi Arabia; 4 Medicine Faculty, Universidad Autonoma de Nuevo Leon, Mitras Centro, Monterrey 64460, Mexico; 5 Department of Pathology and Laboratory Medicine, King Faisal Specialist Hospital and Research Center, P.O. Box 3354, Riyadh 11211, Saudi Arabia

**Keywords:** cord blood, bone marrow, HSC/HPC, hematopoietic, proteomics and gene expression, CD34

## Abstract

**Background/Objectives:** We aimed to identify the molecular signatures of primitive CD34^+^ and CD34^−^ hematopoietic stem/progenitor cell (HSC/HPC) subsets in cord blood and bone marrow samples. **Methods:** CD34^+^ and CD34^−^ HSC/HPC subsets from cord blood and bone marrow were characterized using flow cytometry, real-time PCR, and proteomic analysis to evaluate their phenotypic and molecular profiles. **Results:** Our findings revealed a significantly higher percentage of Lin^−^CD34^−^CD38^Low/−^ (−/−) cells than of Lin^−^CD34^+^CD38^Low/−^ (+/−) cells in cord blood. Aldehyde dehydrogenase levels were significantly lower in (−/−) than in (+/−) cells. Clonogenic ability was lower in (−/−) than in (+/−) cells. However, CD34^−^ cells exhibited potent megakaryocyte/erythrocyte differentiation ability. Importantly, the HSC/HPC subsets expressed pluripotency or stemness genes (SOX2, Nanog, and OCT4); however, OCT4 expression significantly increased in (−/−) compared with (+/−) cells. We identified 304 proteins in the HSC/HPC subsets—85.6% had similar expression patterns in the two subsets; only 14.4% were differentially expressed between (−/−) and (+/−) cells. This implies their comparability at the protein level. Certain proteins were implicated in cellular-development-, gene-expression-, and embryonic-development-related signaling networks. **Conclusions:** Distinct biological and functional characteristics were observed between (−/−) and (+/−) HSC/HPC subsets. Some of the identified proteins may be novel HSC/HPC subsets markers for clinical applications after validation.

## 1. Introduction

Hematopoietic stem/progenitor cell (HSC/HPC) transplantation has revolutionized the treatment of refractory blood diseases, offering curative potential for many patients. HSC/HPCs can be derived from peripheral blood, bone marrow (BM), or umbilical cord blood (CB). Despite the advantages of CB as an HSC/HPC source, its limited quantity restricts its clinical use, particularly in adults. In Saudi Arabia, over 6184 hematopoietic stem cell transplants (HSCTs) were performed between 1984 and 2016, with 5340 cases executed at King Faisal Specialist Hospital and Research Center (KFSH&RC), one of the largest allogeneic HSCT programs globally [1].

HSC/HPCs arise from a rare pool of HSCs, comprising 0.05–0.10% of the BM, CB, or fetal liver [2,3]. These cells are defined by their lifelong ability to self-renew and their potential to differentiate and repopulate the BM of severe combined immunodeficient (SCID) mice [4,5,6,7,8]. Recent advances in genomics and proteomics, enabling single-cell resolution studies on a multi-omics scale, have unveiled the biology of primitive HSC/HPCs. These findings carry significant implications for precision medicine and basic research, as they have shown that primitive HSC/HPCs, along with BM microenvironmental cells, respond to immune-cell-derived stress signals [9,10].

Surface markers like CD34 have traditionally been used to identify and isolate HSC/HPC subsets. Lin^−^CD34^+^CD38^Low/−^ cells (+/−) are commonly recognized as primitive HSCs/multipotent progenitors [11,12]. While the exact function of CD34 is unclear, it is considered essential for the differentiation, adhesion, and proliferation of HSC/HPCs in culture [13,14,15,16]. The classification of human HSC/HPC subsets was facilitated by the sorting of (+/−) cells; however, the definition of HSC/HPC based only on cell surface phenotype is uncertain due to the significant heterogeneity of membrane antigens and their expression levels [17]. Furthermore, the identification of Lin^−^CD34^−^CD38^Low/−^ cells (−/−), which exhibit hematopoietic reconstitution and stem cell properties, challenges the conventional reliance on CD34 for defining HSCs [8,15,16,18,19,20]. Emerging evidence indicates that (−/−) cells play an essential role in hematopoiesis and may offer therapeutic potential. For instance, studies have reported better outcomes with whole BM transplantation compared to sorted (+/−) cells, suggesting that (−/−) cells contribute to improved engraftment and clinical results [8,15,16,18,19,20]. This highlights the clinical significance of (−/−) cells, underscoring their pivotal role in prognosis and therapeutic applications. Previously, we differentiated dendritic cells from (+/−) and (−/−) HSC/HPCs derived from CML patients and healthy BM donors for potential cell therapy applications [21]. The superior ex vivo expansion of (−/−) cells highlights their promise for clinical-grade HSC/HPC applications. However, their characterization remains challenging due to the lack of specific markers, although murine Lin−c-kit+Sca-1+CD34^low/−^ cells exhibit long-term lymphohematopoietic potential [22,23]. Lin^−^CD34^−^CD38^Low/−^ (−/−) HSC/HPCs in humans CB, have been identified via intra-BM injection [24], while a distinct class of HSC/HPC side population (SP) cells expressing low or undetectable CD34 was reported in both humans and animals [5,6,25,26].

Despite their potential, the lack of definitive markers for (−/−) HSC/HPCs has hindered their characterization and clinical application. Techniques such as aldehyde dehydrogenase (ALDH) activity assays and side population (SP) analysis have facilitated the identification of primitive HSCs. High ALDH expression is associated with (+/−) HSC/HPCs, yet limited studies have examined its role in (−/−) subsets [27,28,29,30,31]. Markers like CD133 and CD93 have also been proposed, but their inconsistent expression patterns underscore the heterogeneity of (−/−) HSC/HPCs and the need for further investigation [19,26].

The absence of specific markers limits their study, underscoring the necessity to uncover molecular signatures and identify distinct markers for isolating primitive HSC/HPCs. To address these gaps, this study investigates the functional and molecular properties of (+/−) and (−/−) HSC/HPC subsets using gene expression and proteomic profiling. We hypothesize that a systematic approach employing advanced technologies can identify unique molecular signatures and uncover universal markers to clarify the hierarchical structure of HSC/HPC subsets. These findings could have significant implications for basic research and clinical transplantation, ultimately enhancing the therapeutic potential of HSC/HPCs.

## 2. Materials and Methods

### 2.1. Donors, Human CB/BM Collection, and Cell Isolation

A total of 60 CB samples were collected during the delivery of pregnant women. In total, 10 BM samples allocated from leftover units for routine HSCTs were included in this study. Informed consent was waived, because the Blood Bank would have discarded the collected CB samples due to low total number of nucleated cells or small sample volume. A Ficoll Hypaque gradient (1.077 g/mL density; Pharmacia, NJ, USA) was used to isolate cord blood mononuclear cells (CBMCs). Cell populations sorted with flow cytometry were used for RNA and protein isolation to determine the profiles of stemness-related genes and global protein expression. This study was approved by the Office of Research Affairs and the Research Ethics Committee (ORA & REC) of King Faisal Specialist and Research Center. A permit was obtained from Research Advisory Council (RAC#2140003). The confidentiality and human rights of all subjects were respected.

### 2.2. Immunophenotyping by Flow Cytometry

Samples were collected on day 0. CB was stained as described, with minor modifications [21,32]. In brief, CB samples were washed with PBS (pH 7.4) supplemented with 2 mM EDTA and 0.5% FBS (Gibco, Waltham, MA, USA), incubated for 60 min at 4 °C with ABCG2 polyclonal antibodies, and washed. Then, the cells were incubated for 20 min with APC-conjugated, phycoerythrin-conjugated, or APC-cy-7-conjugated antibodies (anti-CD3, -CD4, -CD8, and -CD45; Becton Dickinson, Franklin Lakes, NJ, USA, or anti-CD27; Dako, Glostrup, Denmark). Matching isotype mAbs conjugated with the appropriate fluorochromes were used as controls. Cells were washed twice with cold PBS and resuspended in FACS buffer. A separate tube containing unstained cells was stained with DAPI and PI to test for viability, and CBMCs were gated to exclude dead cells and debris. Samples were acquired with an LSRII flow cytometer, and data analysis was performed with BD FACSDiva™ software v8.0.1 (Becton Dickinson, Mountain, View, CA, USA).

### 2.3. Cell Sorting and Isolation of Lin− Cell Subsets

A cocktail of FITC-conjugated antibodies against lineage-committed antigens (Lin: CD2, CD3, CD11b, CD14, CD15, CD16, CD19, CD56, CD123, and CD235a) was used. The cells were stained with PerCP-Cy 5.5-conjugated CD34 and phycoerythrin-conjugated CD38 antibodies, followed by negative sorting using BD FACSAria Cell Sorter and FACSDIVA software (BD Biosciences, San Jose, CA, USA). Data were analyzed with FACSDIVA software (Becton Dickinson). Initially, the cells were incubated at 4 °C with a primary antibody labeled with biotin. The purity of the recovered Lin− cells reached >98%. Then, (+/−) and (−/−) HSC/HPCs samples were collected and processed, as described [21]. The expression of the ALDH substrate (ALDEFLUOR assay; Stem Cell Technologies Vancouver, Vancouver, BC, Canada) was detected in addition to three hematopoietic antigens (CD34, CD38, and CD45), as described [31]. The exclusion of dead cells was based on the addition of DAPI (1 μg/10^6^ cells; Beckman Coulter, Franklin Lakes, NJ, USA).

### 2.4. RNA Isolation, cDNA Synthesis, and RT-PCR

Total RNA was extracted from sorted Lin^−^CD34^−^CD38^−^ and Lin^−^CD34^+^CD38^−^ cells using RNAeasy Mini Kit (Qiagen, Valencia, CA, USA), according to the manufacturer’s instructions. RNA was precipitated from the aqueous phase with ethanol and suspended in water. The quality of RNA was determined through 1.5% agarose gel electrophoresis (120 V for 20 min), OD 260/280 ratio analysis, and NanoDrop (Thermo Fisher Scientific, Waltham, MA, USA) analysis. The cDNA was synthesized using Superscript RT-PCR System (Invitrogen GmbH, Karlsruhe, Germany), following the manufacturer’s protocol. The cDNAs were amplified using primers for OCT4, NANOG, and SOX2. The primers were designed using Primer 3 (version 4.0) <http://primer3.ut.ee/> (accessed on 31 December 2012). Gene Runner (version 3.05), and Perl Primer (version v1.1.20) software (Table 1). The RT-PCR conditions were as follows: 94 °C for 15 s, 55 °C for 30 s, and 68 °C for 1 min. The PCR products were resolved on 1.5% agarose gel (120 V for 20 min) with DNA Molecular Weight Marker XIV (100 bp ladder) (Sigma, St. Louis, MO, USA).

### 2.5. Analysis of the Gene Expression Profiles of Sorted (+/−) and (−/−) HSC/HPCs by Real-Time PCR

Gene expression and global protein profiles were evaluated. In brief, (+/−) and (−/−) HSC/HPCs were sorted, and RNA or total proteins were extracted from both CB and BM samples. Gene expression was evaluated by real-time PCR, and proteomics was performed using MS as described below. The expression of stemness genes was quantified using real-time PCR (qRT-PCR) with triplicates on 7500 Fast Real-Time PCR System (Applied Biosystems, Carlsbad, CA, USA), using HotStart-IT Taq Master Mix (Thermo Fisher Scientific, Waltham, MA, USA) and primers designed for OCT4, NANOG, SOX2, and GAPDH as internal controls (Table 1). Gene expression data were analyzed using the 2^−ΔΔCt^ method to estimate relative fold-change as described previously [33]. After each run of PCR, the melting curve was analyzed to confirm the presence of a single band and no artifacts of primer dimers. Real-time PCR data were presented as mean of triplicates ± SD of three independent experiments.

### 2.6. Proteomics

The protein composition of the sorted cells (Lin^−^CD34^−^CD38^−^ and Lin^−^CD34^+^CD38^−^) from CB and BM samples was compared. The analysis included individual samples as a reflection of three biological replicates, and combined or pooled sample cohorts ran multiple times as a reflection of analytical replicates.

#### 2.6.1. Sample Preparation and Protein Extraction

Cell pellets were cautiously thawed and washed in PBS with a cocktail of protease inhibitors (benzamidine and PMSF). Approximately 100–500 µL of RapiGest lysis buffer (Waters, UK) and protease inhibitors were added to each vial. The vials were placed on a shaker at 1000 rpm at room temperature for 3 h. Centrifugation was performed at 13,000 rpm for 15 min to remove insoluble debris. Bio-Rad Protein Assay (Bio-Rad, Hercules, CA, USA) was used to estimate protein content.

#### 2.6.2. Sample Quality Control by 2-DE Electrophoresis

Approximately 75 μg of total solubilized protein from each sample was dissolved in 350 μL of rehydration buffer (2% [*v*/*v*] IPG-buffer 3–10 linear) and loaded onto 11 cm IPG-Strip 3–10 Nonlinear Gel (Bio-Rad, Hercules, CA, USA) to ensure improved resolution and overview of the protein spots across the chosen pH window. PROTEAN IEF System was used to maintain isoelectric focusing. Second-dimension separation was done using Criterion Mini Precast Gels (Bio-Rad, Hercules, CA, USA). The separated proteins were visualized via Cypro Ruby fluorescent staining (Invitrogen Molecular Probes, Eugene, OR, USA). Typhoon Trio Imager (GE, Boston, MA, USA) was used to scan the gels. Data were analyzed using Progenesis Same Spots software (version 7.1.0, Nonlinear Dynamics, Newcastle upon Tyne, UK). Normalized total integrated density or volume was used to measure polypeptide quantities.

#### 2.6.3. Protein in Solution–Digestion Prior to LC–MS Analysis

Aliquots (100 µg) of complex protein mixtures were collected following the concentration and normalization of protein samples. Proteins were denatured in 0.1% RapiGest SF (Waters, Manchester, UK) (one vial diluted in 1000 µL of 50 mM ammonium bicarbonate) at 80 °C for 15 min, reduced in 10 mM DTT at 60 °C for 30 min, and alkylated in 10 mM iodoacetamide for 40 min at room temperature in the dark. Trypsin-digestion of the prepared samples was performed at a 1:50 (*w*/*w*, [1 µg/µL trypsin concentration]) enzyme:protein ratio overnight with gentle shaking at 37 °C. The reaction was quenched with 4 µL of 12 M HCl and incubated at 37 °C for 15 min, followed by centrifugation at 13,000 rpm for 10 min. Aqueous 0.1% formic acid was used to dilute the samples to 1 µg/µL prior to LC/MS analysis. Yeast alcohol dehydrogenase (P00330) was used to spike all samples, as internal standard, at 200 fmol per injection to allow for absolute quantitation.

#### 2.6.4. Protein Identification Using LC/MSGS

We used a one-dimensional nano ACQUITY liquid chromatographer coupled with a Synapt G2 high-definition mass spectrometer (Waters, Manchester, UK), to generate protein expression profiles. The instrument settings before analysis were optimized on the tune page as described [34,35]. In brief, leucine enkephalin (2 ng/µL) was used for the detector set up, and mass calibration was performed with 500 fmol [Glu]1-Fibrinopeptide B (GluFib, 875.843 Da) using MassLynx IntelliStart. The other MassLynx tune page parameters included a capillary voltage of 3 kV, sample cone of 50 V, extraction cone of 5 V, and source temperature of 80 °C. All analyses were performed using Trizaic Nano Source (Waters, Manchester, UK) ionization in the positive ion mode nanoESI. Equal amounts of 3 µg protein digest/sample were loaded onto the column. Each sample was infused using automated sample loading, with the mobile phase consisting of A1 (99% water, 1% acetonitrile + 0.1% formic acid) and B1 (acetonitrile + 0.1% formic acid) at a sample flow rate of 0.450 µL/min. Data-independent acquisition (MS)/iron mobility separation experiments were performed and acquired over a short range of *m*/*z* 50–2000 Da, with a total gradient acquisition time of 120 min, as described [34,35]. All samples were analyzed in triplicate runs, and data were acquired using MassLynx (version. 4.1, SCN833, Waters, Manchester, UK), run in resolution and positive polarity modes. Automated data processing and database searching were performed using Progenesis QI for proteomics V3 (Waters, Manchester, UK/Nonlinear Dynamics, Newcastle/, UK). The generated peptide masses were searched against the UniProt protein sequence database, using Progenesis QI for protein identification (Waters, UK).

### 2.7. Colony-Forming Assay

Hematopoiesis of the Lin^−^CD34^−^CD38^−^ (−/−) and Lin^−^CD34^+^CD38^−^ (+/−) fractions was evaluated using the standard CFU assay. In summary, cultures from the two HSC/HCP subsets were initiated in Methocult or RPMI 1640 supplemented with the rhFlt3 ligand, recombinant human (rh) thrombopoietin (10 ng/mL), and rh stem cell factor. Cells from the HSC/HPC subsets were cultured and evaluated daily for 14 d, starting from days 0–14, for CFU formation. The data presented are exclusively for CB, with BM CFU assays excluded but considered indicative of CB samples.

All experiments were performed using 1000 HSCs/plate. On day 14, counting was conducted for both HSC subsets, and CFU counts reflected the number of colonies per plate. Only a few megakaryocyte-containing colonies (GEMMs) were found. On day 14, the colonies were counted and assessed both quantitatively and qualitatively, with an emphasis on their morphologies. Cultures were performed in duplicate and incubated at 37 °C in a humidified incubator with 5% CO_2_. An inverted microscope was used to assess colony morphology after 10–14 d. The number of colonies and CFU subpopulations generated from (+/−) and (−/−) HSC/HPC subsets were analyzed using medians and interquartile ranges and compared using the Mann–Whitney test.

### 2.8. Statistical Analysis

Depending on data distribution, means and SD or medians and interquartile ranges summarized continuous data, whereas frequencies and percentages characterized categorical data. Surface marker expression and protein composition were compared using paired Student’s *t* test and Wilcoxon signed-rank test, based on data distribution. Protein compositions were analyzed using ANOVA or the Kruskal–Wallis test, accounting for multiple comparisons. Median confidence intervals were established, as noted above, and *p* < 0.05 was considered statistically significant. Cluster hierarchy and proteome profiling analyses used Log10 ratios. IBM SPSS Statistics 24 was used for statistical analysis.

To analyze proteomics data, we used an algorithm in the license-based Progenesis QI for proteomics (Nonlinear Dynamics, Newcastle/Waters, UK). We extracted significant changes in differential expression between the sample pairs compared. To address multiple testing and false discovery rate, we used adjusted *p*-value or *q*-value, as described [34,35,36]. We applied multivariate data analysis to identify only statistically significantly regulated proteins of normalized label-free quantifications of protein abundance. Moreover, “Hi3” absolute quantification was performed with a known protein as internal standard for the absolute amount of each identified protein. The identified proteins were filtered to show only those with confidence identifications with ≥2 peptides identified and statistically (ANOVA) significantly altered proteins (*p* ≤ 0.05) and a fold-change of >1.5.

## 3. Results

### 3.1. Kinetics of the HSC/HPC Subsets in CB and BM Samples

**Figure 1:**
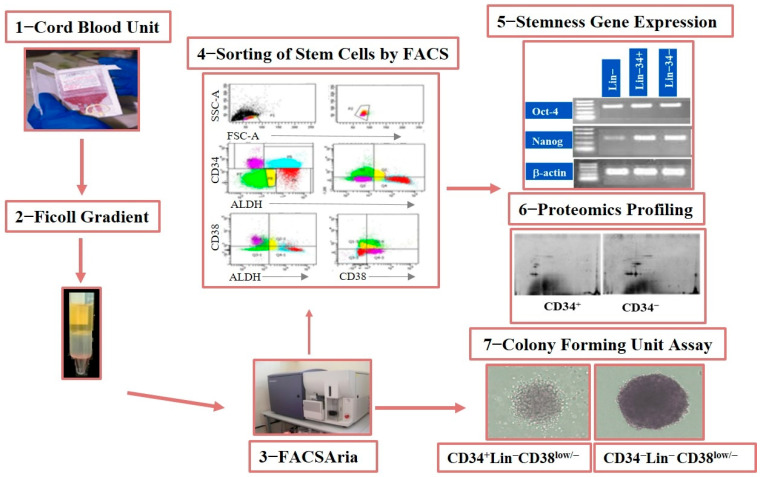
Schematic of the experimental layout. Mononuclear cells were isolated using Ficol from CB samples obtained from women who underwent delivery. HPC/HSCs were sorted by FACS, collected, and functionally characterized using CFU analysis. HPC/HSCs were molecularly characterized through gene expression and proteomics analyses using real-time PCR and label-free quantitative liquid chromatography–tandem mass spectrometry (LC–MS/MS), respectively. The presence of Lin^−^CD34^−^CD38^Low/−^ (−/−) and Lin^−^CD34^+^CD38^Low/−^ (+/−) subgroups was assessed using flow cytometry. Their kinetics and molecular and cellular profiles were then compared (Figure 2).

**Figure 2:**
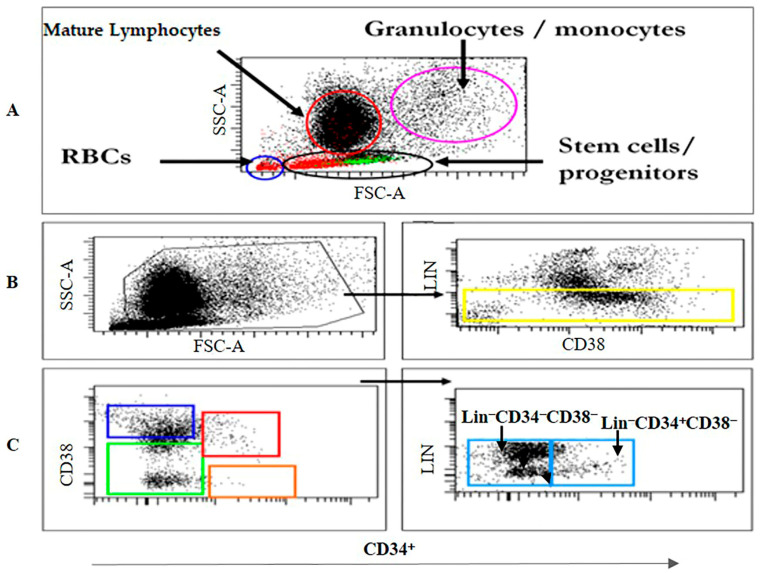
Gating strategy. (**A**) Definition of primitive HSC/HPCs in a representative CB sample. (**B**) Gating of Lin^−^CD34^+^CD38^Low/−^ cells followed by gating of CD34 in combination with CD38 identified four populations: Lin^−^CD34^−^CD38^+^ (blue box), Lin^−^CD34^+^CD38^+^ (red box), Lin^−^CD34^−^CD38^Low/−^ (green box), and Lin^−^CD34^+^CD38^Low/−^ (orange box). (**C**) The study focused only on two populations depicted in (**C**) as follows: Lin^−^CD34^−^CD38^Low/−^ (−/−) and Lin^−^CD34^+^CD38^Low/−^ (+/−) cells.

Approximately 60 CB samples were collected from women who reached full term and delivered at local hospitals in the Riyadh area. Twenty samples were discarded due to low quality or presence of blood clots or contamination. Finally, 40 CB samples were used Table S1. HSC/HPCs were isolated and subjected to flow cytometry, gene expression profiling, proteomics, and colony-forming unit (CFU) analysis (Figure 1 and Figure 2).

**Figure 3:**
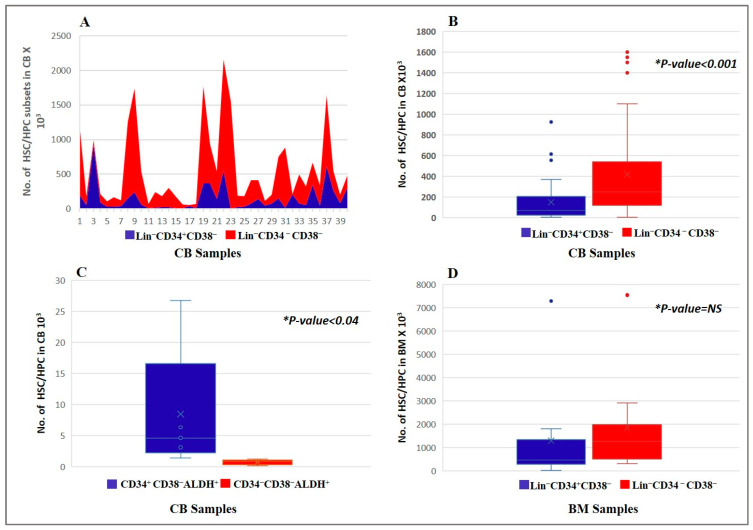
Phenotypic characterization of the HSC/HPC subsets in CB and BM samples. (**A**) The levels of Lin^−^CD34^+^CD38^Low/−^ and Lin^−^CD34^−^CD38^Low/−^ fractions in the same donor in 40 sorted CB samples. (**B**) The difference in the numbers of sorted Lin−CD34^−^CD38^Low/−^ (+/−) and Lin^−^CD34^+^CD38^Low/−^ (−/−) cells in 40 CB samples is significant (*p* = 0.001). (**C**) The difference in the numbers of sorted Lin^−^CD34^−^CD38^Low/−^ ALDH+ and Lin^−^CD34^+^CD38^Low/−^ ALDH+ cells is significant (*p* = 0.04). (**D**) The difference in the numbers of the two HSC/HPC subsets in 10 BM samples is not significant (*p* = 0.06).

Interestingly, our analysis revealed that (+/−) and (−/−) HSC/HPCs were present in CB samples in variable proportions. Notably, the median of cells expressing (+/−) (67.000, IQR range: 1.030−926.000 × 10^3^) was significantly lower than of those expressing Lin^−^CD34^−^CD38^Low/−^ (−/−) (249.500, IQR: 1.924–1600.000 × 10^3^) (*p* < 0.001; Figure 3A,B). By contrast, a reduced median percentage of ALDH marker was recorded in (−/−) cells (0.8, IQR: 0.30–1.1%) compared with (+/−) cells (4.6, IQR: 2.2–16.5%). This difference was statistically significant (*p* = 0.04; Figure 3C). The higher upper limit observed in CB whiskers is attributed to biological variability among donors, emphasizing the heterogeneity in marker expression. The same trend was observed in BM samples. The median of sorted (−/−) cells (1255.000; 310.000−7550.000 × 10^3^) was higher than that of (+/−) cells (470.000; 11.000−7300.000 × 10^3^), although the difference was not statistically significant (Figure 3D). The stemness features of the two HSC/HPC subsets were validated in a number of CB and BM samples based on the presence of SP (Figure S1). However, further studies are needed to determine whether the (+/−) HSC/HPC subset is more primitive than the (−/−) subset.

### 3.2. Gene Expression Profiles of CD34^+^ and CD34^−^ Cells in CB and BM Samples

**Figure 4:**
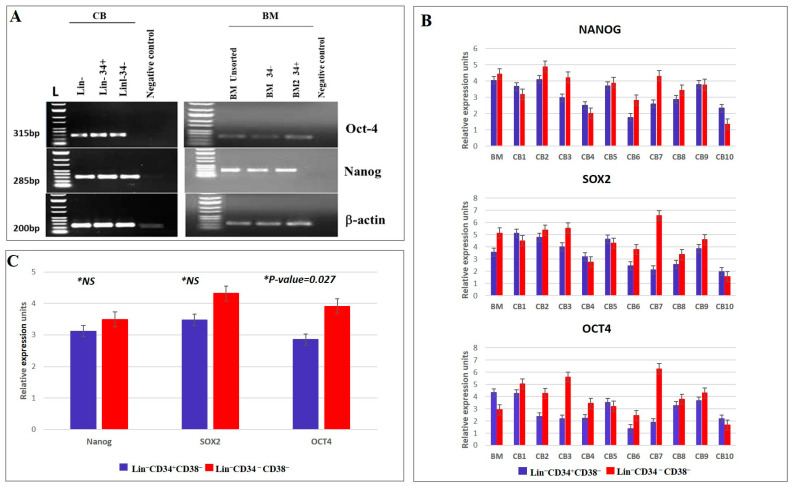
Gene expression profiles of *Nanog*, Sox2, and *OCT4* in sorted and unsorted HSC/HPCs in CB and BM samples. (**A**) Relative gene expression of *Nanog*, Sox2, and *OCT4* in sorted and unsorted BM and CB cells, L−100 bp ladder. (**B**) Means of relative gene expression profiles of three transcription factors associated with stemness (*Nanog*, *SOX2*, and *OCT4*) were measured in primitive HSC/HPCs (Lin^−^ CD34^−^ vs. Lin^−^CD34^+^) from ten cord blood samples. (**C**) Means of relative gene expression of *Nanog*, *SOX2*, and *OCT4*. Only difference in Oct4 reached statistical significance between the two subsets (*p* = 0.027). Three replicates were used for RT-PCR. (Please see un-cropped representative raw UV images of RT-PCR gels for two samples images in Supplementary Figure S2).

Transcriptional profiling of stem cells has identified genes that regulate stemness, such as ability to self-renew and maintain an undifferentiated state. The (−/−) and (+/−) HSC/HPC subsets were sorted from both CB and BM samples, and their RNA was extracted. The gene expression profiles were determined through real-time PCR. Both HSC/HPC subpopulations expressed pluripotency/stemness genes, such as Nanog, SOX2, and Oct4 (Figure 4A,B). Median Nanog expression was 2.595 (IQR: 2.092–3.687) and 3.902 (IQR: 2.628–4.253) in (+/−) and (−/−) cells, respectively. Median SOX2 expression was 3.237 (IQR: 2.351–4.713) for the (+/−) group and 4.403 (IQR: 2.241–5.410) for the CD34^−^ group. Median Oct4 expression was 2.218 (IQR: 1.916–3.450) for the (+/−) group and 3.879 (IQR: 3.0929–5.181) for the (−/−) HSC/HPC group. The distribution of Oct4 between (+/−) and (−/−) subgroups showed statistically significant difference (*p* = 0.027; t = 2.643). However, differences in the distribution of Nanog and SOX2 between (+/−) and (−/−) groups were not statistically significant (*p* = 0.2 and *p* = 0.1, respectively). These results indicate that both fractions have comparable primitive stemness features, as shown in Figure 4C.

### 3.3. Global Protein Expression Profiles of CD34^+^ and CD34^−^ Cells in CB and BM Samples

**Figure 5:**
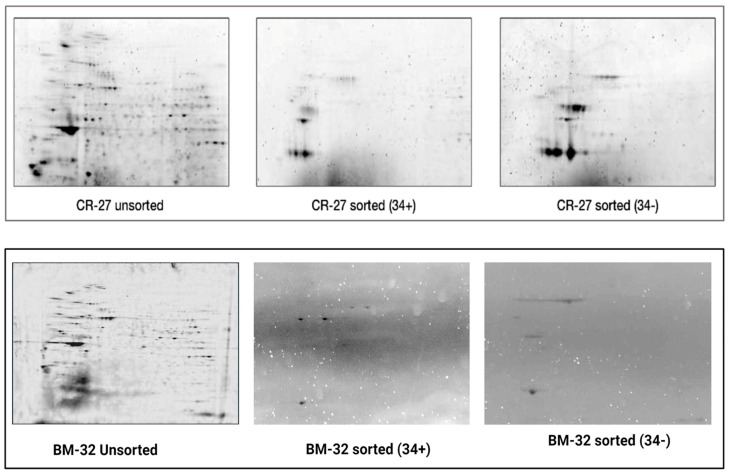
Representative 2-DE images from unsorted, CD34+, and CD34^−^ cord blood HSC/HPCs. Marked differences in the total number of protein spots were observed in unsorted vs. CD34 HSC/HPC sorted samples. A high degree of similarity was observed in the total number of resolved spots in both CD34^−^ and CD34^+^ HSC/HPC subsets. CR27 and BM-32 denote the coded identification numbers of participants from whom the samples were collected. (Please see un-cropped representative 2-DE gel images in Supplementary Figure S3).

Proteomics analysis was performed using mass spectrometry (MS). Due to the intrinsic limitations of the high-throughput MS-based analysis used, 2-D gel electrophoresis was first performed (using cells derived from CB and BM) to access individual variability of protein expression across HSC/HPC cell populations. The qualitative and quantitative features of the 2-DE gels were used as a form of quality control of all samples pooled as a cohort prior to MS analysis of BM-derived cells, as described [37]. Only samples with a high degree of similarity judged by 2-DE signatures were pooled together. Samples with marked variations were excluded from pooling. Representative images derived from each of the sample cohorts of CD34^+^ and CD34^−^ HSC/HPC CB are shown in Figure 5.

### 3.4. Protein Expression Changes and Annotation of the Identified Proteins Using Knowledge-Based Ingenuity Pathway Analysis

**Figure 6:**
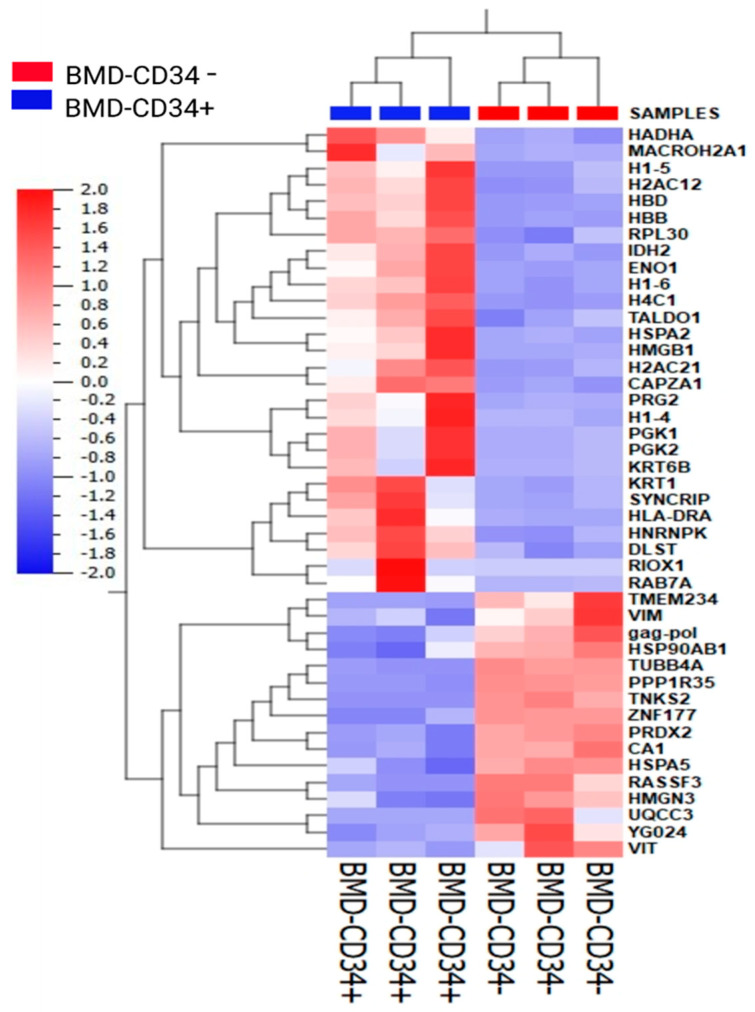
Hierarchical cluster analysis using the 44 differentially expressed proteins between samples labeled with solid color red representing BMD CD34− and solid blue BMD CD 34+. Heat map illustrates the technical replicates of relative quantities of pooled proteins from each cohort of bone marrow (BMD) sorted CD34+ and CD34− cell samples. A gradient of colors is used to represent the scaling range of expression values, with red and blue indicating degree of up- and downregulation, respectively. The image was generated using Qlucore Omics Explorer version 3.7 (Lund, Sweden) (https://qlucore.com) (accessed on 12 July 2024).

Using label-free quantitative expression proteomics analysis, we identified 304 proteins in the (+/−) and (−/−) BM-derived HSC/HPC subsets. Most of the expressed proteins (260/304 identified proteins; ~85.6%) were similarly expressed in the two HSC/HPC fractions. Only 44 proteins (~14.4%) were differentially expressed in (+/−) and (−/−) HSC/HPCs (Table S2). This finding indicates that the two subsets are fairly comparable at the protein level. The 44 differentially expressed proteins were further analyzed for their molecular functional annotation using the knowledge-based Ingenuity Pathway Analysis (IPA, Qiagen, MD, USA). Only 30/44 differentially expressed proteins were mapped in the IPA database. Characteristics including fold-change and cellular localizations are outlined in Table 2. Protein–protein interactions of some proteins had direct or indirect connections with known stem cell markers, such as NANOG and STAT3 (see network images in Figure S4A,B). These proteins are involved in gene expression, protein synthesis, and embryonic development pathways. The change in expression of the 44 proteins was subjected to unsupervised hierarchical cluster analysis (see graph in Figure 6).

### 3.5. Functional Analysis—Colony Formation

**Figure 7:**
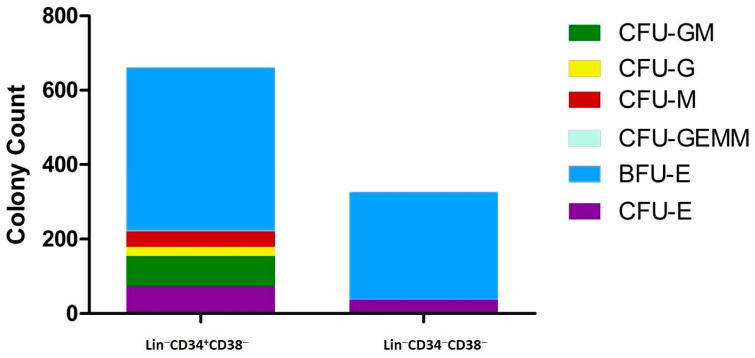
Total colony-forming units (CFU) produced by (+/−) and (−/−) HSC/HPC subsets in eight CB samples. All experiments were performed using 1000 HSCs/plate and on day 14. Counting was conducted for both HSC subsets. CFU counts reflect the number of colonies per plate. BFU-E and CFU-E colonies were measured in both subsets. The CD34^+^ fraction produced CFU-E, BFU-E, CFU-G, CFU-M, CFU-GM, and CFU-GEMM colony lineages. The difference in the number of colonies between the two subsets is not statistically significant (*p* > 0.05, *n* = 8).

The hematopoietic potential of both (+/−) and (−/−) HSC/HPC subsets was assessed using the CFU method. Colonies were quantified under a microscope after 14 days of culture. The (+/−) HSC/HPC subset gave rise to several hematopoietic cell lineages, including CFU-erythroid (CFU-E), burst-forming unit-erythroid (BFU-E), granulocytes (CFU-G), macrophages (CFU-M), granulocyte-macrophages (CFU-GM), and erythrocytes, monocytes, and megakaryocytes (CFU-GEMM). The total number of colonies was 1187 with a median of 41 (IQR: 1–104). Our results of CFU in CB are summarized in Table 3, which shows that the (+/−) HSC/HPC subset generated more colonies, totaling 661 with a median of 58 (IQR: 34, 140.3), compared with the (−/−) HSC/HPC subset, which produced 326 colonies with a median of 1 (IQR: 1, 64.7) (Table 3, Figure 7 and Figure S5A,B). The number of colonies formed by the two HSC/HPC subsets was not statistically significantly different (*p* > 0.5) (Figure 7). Erythroid progenitor cells, including CFU-E and BFU-E, were produced by both positive and negative subsets (Figure 7 and Figure S5A,B). It is worth noting that the (−/−) HSC/HPC subset had the ability to differentiate into both megakaryocytes and erythrocytes (Table 3).

## 4. Discussion

The ability of stem cells to differentiate into multiple types of committed cells while maintaining their stemness makes them attractive targets for cell-based therapy. HSCTs have been utilized in regenerative therapy for the past six decades [38]. However, studies have focused on (+/−) cells, neglecting the more primitive (−/−) subset, which remains poorly characterized. In fact, most of our knowledge on hematopoiesis comes from animal or xenotransplantation studies using HSC/HPC enrichment based on CD34 expression [11,12,39]. Reports on (−/−) HSC/HPC subsets in humans are scarce and inconsistent, because different markers have been used to enrich early stem and progenitor HSC/HPC subsets in CB samples [26,40]. Few studies have performed comprehensive phenotypic, molecular, and functional analyses of both (+/−) and (−/−) HSC/HPC subsets in human CB samples, and data on (−/−) HSC/HPC subsets in human BM samples are rare or lacking [26,40]. Due to its lack of positive markers, the (−/−) HSC/HPC fraction is enriched based on the absence of CD34, resulting in a heterogeneous population of HSC/HPCs with an undefined molecular makeup and limited therapeutic utility. Our study addressed this gap by conducting a comprehensive analysis and combining phenotypic, molecular, and functional assessments of both (+/−) and (−/−) fractions in CB, and to a lesser extent, BM samples. Despite the limited number of BM samples, our results underscore the relevance of (−/−) HSC/HPCs, presenting insights into their biology and function. The presence of CD34^−^ cells in both CB and BM, with their stemness features highlighted through molecular and functional assays, supports their potential clinical applications. Notably, our study revealed that (−/−) cells may surpass (+/−) cells in certain aspects, hinting at their therapeutic advantages. Notably, (−/−) cells have been described both in humans and animals [8,20,22,26]. Their clinical significance is supported by the notion that the clinical outcome is sometimes not correlated with the quantity or quality of transplanted (+/−) cells. The clinical significance of (−/−) cells is supported by the finding that transplanting whole BM compared with sorted (+/−) cells improved clinical outcomes. ALDH is a stem cell marker that is believed to regulate the differentiation of HSCs. ALDH is expressed in various stem cell types in different tissues [41,42]. An essential aspect of our investigation is the identification of ALDH as a marker associated with (−/−), emphasizing the heterogeneous expression of ALDH in different fractions [43]. Studies have reported that although CD34 expression was upregulated in (−/−) cells in vitro [16], the SCID repopulation capacity of (−/−) cells was much lower than that of (+/−) HSC/HPCs [44], indicating that induction does not produce genuine (+/−) cells. We assessed the in vitro differentiation potential of primitive (+/−) and (−/−) subsets into lineage-specific cells. CD34+ HSCs formed colonies of mixed hematopoietic stem cell (CFU mixed), CFU-GM, and BFU-E progenitors. Alternatively, (−/−) exhibited significant capacity for differentiating into megakaryocytes and erythrocytes, evidenced by CFU-E and BFU-E progenitors. These results are consistent with those of Sonoda et al. [40]. Several reports have suggested that the (+/−) HSC/HPC subset often possesses a higher hematopoietic potential, highlighting the complexity and versatility of HSC/HPCs [8,19,24,25,44,45,46]. This has led to the recognition of CD34− cells as a new concept in the hierarchy of human HSC/HPCs. They could be more primitive than Lin−CD34+ cells and lie at the apex of human HSC/HPCs [19,40]. Although commonly used assays, such as CFU, are informative for (+/−) cells, our study suggests using additional tests tailored for (−/−) cells to assess their functional capacity, which is consistent with other reports [27,30,31].

To facilitate the selection of (−/−) cells for research and clinical applications, a high-resolution purification method using 18 Lin mAbs was developed by Sonoda et al. [47]. CD133 was more effective than CD34 and could equally detect (+/−) and (−/−) HSC/HPCs in CB for HSCT [19]. Notta et al. showed that CD49f is a specific HSC marker [48]. However, contradictory reports suggest that there may be heterogeneous populations of primitive (−/−) HSC/HPCs. Later, Sonoda et al. identified two positive enrichment markers (CD133 and GPI-80) for CD34− cells [40]. Further investigations are needed to clarify these discrepancies, particularly because the absence of a positive marker for the enrichment of (−/−) HSC/HPCs leads to a waste of the valuable (−/−) HSC/HPC fraction [26].

Adult somatic stem and progenitor cells have several characteristics in common with pluripotent stem cells, such as the ability to develop into various cell types and express specific transcriptional regulators [49,50]. Herein, further evidence for pluripotency came from the observation that pluripotency- or stemness-related genes, including SOX2, Nanog, and OCT4, were expressed in both HSC/HPC subsets (quantified using real-time PCR). Adult somatic stem cells share characteristics with pluripotent stem cells, such as the ability to develop into various cell types and to express specific transcriptional regulators. Studies have shown that pluripotency-related genes, including SOX2, Nanog, and OCT4, are expressed in both subsets of HSC/HPC. These transcription factors are highly expressed in embryonic stem cells, and their absence or reduction is correlated with the loss of pluripotency and self-renewability. Other studies have confirmed these findings [51,52]. Recently, Lee et al. (2023) observed a significant increase in stemness markers and upregulation of stem cell genes, including OCT4, NANOG, SOX2, and TERT, in both leukocytes and tiny embryonic-like stem cells after ex vivo expansion [53]. Their findings showed that optimal culture techniques can enhance pluripotent gene expression and plasticity, representing a significant breakthrough in the field of regenerative medicine.

Proteomics analysis revealed many similarities in protein composition between the two HSC/HPC subsets. Approximately 85.6% of all proteins were similarly expressed in the two fractions, indicating that the two subsets are fairly comparable at the protein level. Network analysis revealed that most of the differentially expressed proteins are involved in different forms of maintenance of stem cells in general and hematopoietic stem cells in particular. Of the proteins identified in this study, IDH2, PGK1, PGK2, peroxiredoxin 2 (PRDX2), PRG2, RAC2, RPL30, and TALDO1 deserve to be highlighted. IDH2 compensated for IDH1 mutations in the maintenance of cell survival under hypoxic conditions [54]. PGK1 increased neurite outgrowth in cells [55]. PRDX2 was involved in maintaining cancer stem cells [56]. RAC2 was involved in interactions with the hematopoietic microenvironment [57]. The roles of the other differentially expressed proteins in stem-cell-related functions remain to be determined. Finally, identifying the molecular signature of the HSC/HPC subsets and their functions is crucial and will direct the discovery of distinct markers, indispensable for the isolation of the most primitive HSC/HPCs. Such a breakthrough will have important implications for cell-based clinical transplantation.

Our study has limitations. There was a scarcity of BM samples, and proteome analysis was challenging due to limited sorted cells. We also acknowledged that there might be some regional peculiarities and ethnic-specific factors such as population genetics, disease prevalence, and environment that can influence proteomic analysis, important to bear in mind when interpreting proteomic data from different populations. This is important especially in maintaining the accuracy of clinical research settings for discovery of accurate biomarkers and therapeutic strategies. However, the samples analyzed in this study were homogeneously Saudi nationals. In any case, our research emphasizes the importance of investigating (−/−) HSC/HPCs. This study recognizes their existence, significance, and stemness and advocates for research to validate shared proteins within HSC/HPC subpopulations, considering the variability between individuals. Unraveling the molecular and functional signatures of (−/−) HSC/HPCs is crucial for advancing cell-based clinical transplantation.

## Figures and Tables

**Table 1:** Primer sequences and product sizes for gene expression analysis.

Gene	Primer	Product Size (bp)
**Oct4**	F GAAGGTATTCAGCCAAACGAC R GTTACAGAACCACACTCGGA	315
**Nanog**	F TGCAAATGTCTTCTGCTGAGAT R GTTCAGGATGTTGGAGAGTTC	285
**Sox−2**	F ATGCACCGCTACGACGTGA R CTTTTGCACCCCTCCCATTT	437
**Human GAPDH**	F GTCAGTGCTGGACCTGACCT R CACCACCATGTTGCTGTAGC	255

**Table 2:** List of the 44 differentially expressed proteins in the CD34 (+/−) and (−/−) BM-derived HSC/HPC subsets.

Accession	Symbol	Entrez Gene Name-Description	IPA Expr *p*-Value Ranking	* Proteomics Expr Fold-Change: CD34^+^/^−^	Location	Type(s)
P00915	CA1	carbonic anhydrase 1	5.29 × 10^−3^	−5115	Cytoplasm	Enzyme
P52907	CAPZA1	capping actin protein of muscle Z-line subunit alpha 1	2.92 × 10^−3^	2442	Cytoplasm	Other
P36957	DLST	dihydrolipoamide *S*-succinyltransferase	6.64 × 10^−3^	2461	Cytoplasm	Enzyme
P16401	H1-5	H1.5 linker histone, cluster member	1.16 × 10^−2^	2595	Nucleus	Other
P22492	H1-6	H1.6 linker histone, cluster member	2.71 × 10^−3^	2787	Nucleus	Other
Q8IUE6	H2AC21	H2A clustered histone 21	1.04 × 10^−2^	3055	Nucleus	Other
P40939	HADHA	hydroxyacyl-CoA dehydrogenase trifunctional multienzyme complex subunit alpha	4.45 × 10^−3^	6879	Cytoplasm	Enzyme
P02042	HBD	hemoglobin subunit delta	1.33 × 10^−3^	3325	Other	Transporter
Q15651	HMGN3	high mobility group nucleosomal binding domain 3	4.94 × 10^−2^	−5861	Nucleus	Other
P61978	HNRNPK	heterogeneous nuclear ribonucleoprotein K	3.36 × 10^−3^	2732	Nucleus	Other
P54652	HSPA2	heat shock protein family A (Hsp70) member 2	8.41 × 10^−3^	3396	Cytoplasm	Other
P48735	IDH2	isocitrate dehydrogenase (NADP(+)) 2	2.04 × 10^−3^	9253	Cytoplasm	Enzyme
P04264	KRT1	keratin 1	1.57 × 10^−2^	4856	Cytoplasm	Other
P00558	PGK1	phosphoglycerate kinase 1	3.06 × 10^−2^	3595	Cytoplasm	Kinase
P07205	PGK2	phosphoglycerate kinase 2	3.06 × 10^−2^	3595	Cytoplasm	Kinase
Q8TAP8	PPP1R35	protein phosphatase 1 regulatory subunit 35	1.90 × 10^−2^	−34,676	Cytoplasm	Other
P32119	PRDX2	peroxiredoxin 2	2.85 × 10^−2^	−8288	Cytoplasm	Enzyme
P13727	PRG2	proteoglycan 2, pro-eosinophil major basic protein	9.77 × 10^−3^	4217	Extracellular Space	Other
P51149	RAB7A	RAB7A, member RAS oncogene family	3.48 × 10^−2^	3120	Cytoplasm	Enzyme
Q86WH2	RASSF3	Ras association domain family member 3	4.71 × 10^−3^	−17,993	Other	Other
Q9H6W3	RIOX1	ribosomal oxygenase 1	5.18 × 10^−3^	1000,000	Nucleus	Enzyme
P62888	RPL30	ribosomal protein L30	6.50 × 10^−3^	3379	Cytoplasm	Other
O60506	SYNCRIP	synaptotagmin binding cytoplasmic RNA interacting protein	2.00 × 10^−2^	3041	Nucleus	Other
P37837	TALDO1	transaldolase 1	1.49 × 10^−2^	3146	Cytoplasm	Enzyme
Q8WY98	TMEM234	transmembrane protein 234	4.12 × 10^−2^	−45,290	Other	Other
Q9H2K2	TNKS2	tankyrase 2	2.18 × 10^−3^	−273,322	Nucleus	Enzyme
P04350	TUBB4A	tubulin beta 4A class IVa	2.14 × 10^−2^	−68,781	Cytoplasm	Other
Q6UW78	UQCC3	ubiquinol–cytochrome c reductase complex assembly factor 3	1.76 × 10^−2^	−168,107	Extracellular Space	Other
Q6UXI7	VIT	vitrin	1.79 × 10^−2^	−8856	Extracellular Space	Other

Note: * Fold-change was calculated and converted to negative values, based on the expression values of the (+/−)/(−/−) HSC/HPC subsets. Positive fold-change values represent upregulation in (+/−) cells compared with (−/−) cells. Negative fold-change values represent upregulation in (−/−) cells compared with (+/−) cells.

**Table 3:** Colony-forming units measured in HSC/HPC subsets (CD34^+^ and CD34^−^).

Factors	Median	Min	Q1	Q3	Max	Median	Min	Q1	Q3	Max	Median	Min	Q1	Q3	Max	*p*-Value
	Whole Group					CD34-					CD34+					
Colonies	41	1	1	104.25	200	1	1	1	65.75	200	58.50	10	34	140.20	199	NS *
CFU-E	0	0	0	1	64	0	0	0	1	35	0.50	0	0	5.50	64	NA^+^
BFU-E	5	0	0	65.75	200	1	0	0	57	200	20.5	0	0	124.20	199	NA
CFU-GM	0	0	0	0	74	0	0	0	0	0	0	0	0	5.25	74	NA
CFU-G	0	0	0	0	25	0	0	0	0	0	0	0	0	0	25	NA
CFU-M	0	0	0	0	42	0	0	0	0	0	0	0	0	0	42	NA
CFU-GEMM	0	0	0	0	1	0	0	0	0	0	0	0	0	0	1	NA

*: not significant (*p*-value = 0.6). +: not applicable; number of observations per group is not sufficient for a statistical test. The insufficient number of observations per group precludes conducting a statistical test. This is because the (−/−) HSC/HPC subset comprises eight samples with a skewed distribution: five samples contain one colony each, while the remaining three samples have 50, 300, and 71 colonies, respectively. For further information, please consult the graph below.

## Data Availability

The data supporting the reported results are saved in secured files in our server and available from the corresponding author upon reasonable request.

## Supplementary Materials

The following supporting information can be downloaded at: www.mdpi.com/article/10.3390/diagnostics15040447/s1, Figure S1: SP cells within HSC/HPCs from three cord blood donors. Density plots obtained from flow cytometry illustrate the identification of viable cells and the identification of the verapamil-sensitive side population (SP) region. Figure S2: un-cropped representative UV images from the gels of two samples one from CB and one from BM. Figure S3: un-cropped representative 2-DE gel images Figure S3A,B: Figure S4: Representation of the Ingenuity Pathway Analysis (IPA) of some of the 44 differentially expressed proteins (+/−) and (−/−) HSC/HPC subsets. (A) Highlighted in pink are some of the identified proteins involved in gene expression, protein synthesis, and RNA damage and repair signaling pathways. Grey represents the other molecules in the IPA database that made up the entire network. (B) Other molecules were implicated in cellular development, and embryonic development signaling pathways. In the core of the network is Nanog and stat3 with other interacting proteins. The network analysis was generated using IPA (https://www.qiagenbioinformatics.com (accessed on 31 December 2012)). Figure S5: Displays representative results of Colony-forming assays from eight CB samples. (A) Both (+/−) and (−/−) HSC/HPC subsets were sorted from 8 CB samples and cultured for 14 days. Colony formation—erythroid progenitor colonies namely burst-forming unit-erythroid (BFU-E) and colony-forming unit-erythroid (CFU-E)—were measured in both CD34^+^ and CD34^−^ subsets. (B) Whereas, CFU-E, BFU-E, CFU-G, CFU-M, CFU-GM, and CFU-GEMM colonies were only produced by CD34^+^ fraction. Table S1: Presents the absolute numbers of CBMCs and the Lin-CD34+CD38 Low/− HSC/HPC to Lin-CD38 Low/− HSC/HPC fractions in 40 CB samples. Table S2. List of 44 differentially expressed proteins in CD34^+^ versus CD34^−^ HSC/HPCs cells.
