# Supplementary material for: Distinct Phenotypic and Molecular Characteristics of CD34− and CD34+ Hematopoietic Stem/Progenitor Cell Subsets in Cord Blood and Bone Marrow Samples: Implications for Clinical Applications
Source: Diagnostics (Basel). 2025 Feb 12;15(4):447. doi: 10.3390/diagnostics15040447 (PMC11853955; doi:10.3390/diagnostics15040447)
Supplement: Supplementary file 1 [file diagnostics-15-00447-s001.zip › Supplementary - 2025.pdf]

Supplementary Information

Distinct Phenotypic and Molecular Characteristics of CD34¯ and CD34+ Hematopoietic Stem/Progenitor Cell Subsets in Cord Blood and Bone Marrow Samples: Implications for Clinical Applications.

Ameera Gaafar[1,2]*, Fatheia Nabeil Hamza[1,2], Rama Yousif[1], Zakia Shinwari[1], Aminah Ghazi Alotaibi[1], Alia Iqniebi[1], Khalid Al-Hussein[1], Amer Al-Mazrou[1], Pulicat Subramanian Manogaran[1], Tusneem Elhassan[2,3], Marcela Marquez-Méndez [4], Mahmood Aljurf[3], Hind Al-Humaidan[5] and Ayodele Alaiya[1]*

[1]Cell Therapy & Immunobiology Department, King Faisal Specialist Hospital & Research Center, P.O. Box 3354, 11211, Riyadh, Saudi Arabia.
[2] Biochemistry & Molecular Medicine Department, Alfaisal University, P.O. Box 3354, 11211, Riyadh, Saudi Arabia.
[3]Cancer Center for Excellence, King Faisal Specialist Hospital and Research Center, Riyadh, Saudi Arabia.
[4]Medicine Faculty, Universidad Autonoma de Nuevo Leon, Mitras Centro 64460, Monterrey, N.L. Mexico.
[5]Department of Pathology and Laboratory Medicine, King Faisal Specialist Hospital & Research Center, P.O. Box 3354, 11211, Riyadh, Saudi Arabia.

*Corresponding authors:

Ameera Gaafar, PhD

Ayodele Alaiya, MB.BS, PhD

Stem Cell & Tissue Re-engineering Program,
King Faisal Specialist Hospital & Research Center,
P.O. Box 3354, 11211, Riyadh, Saudi Arabia.
Phone +114647272; Ext: No. 32537Email: agaafar@kfshrc.ediu.sa; AAlaiya@kfshrc.edu.sa

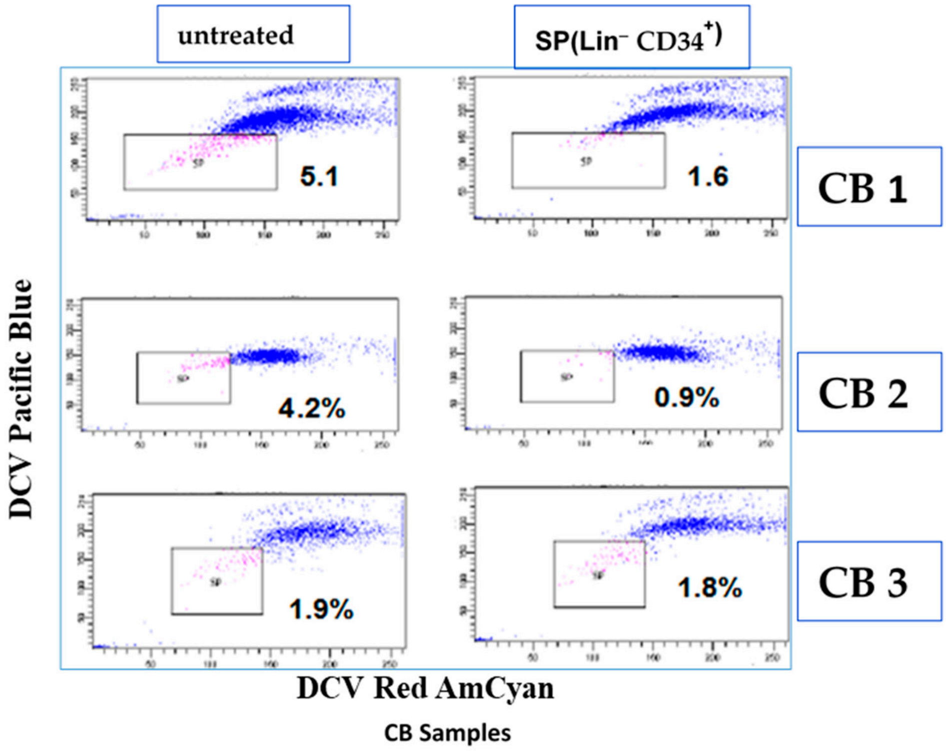

Figure S1. SP cells within HSC/HPCs from three cord blood donors. Density plots obtained from flow cytometry illustrate the identification of viable cells and the identification of the verapamil-sensitive side population (SP) region.

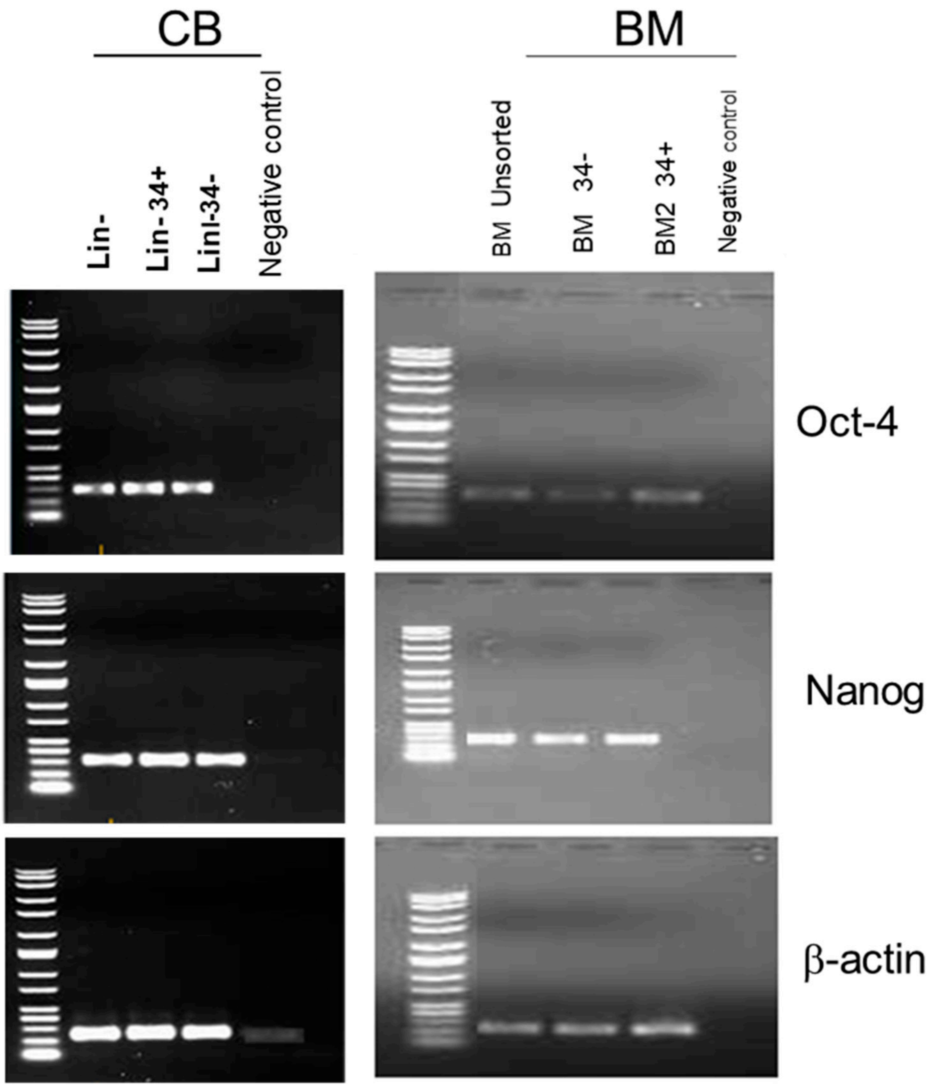

Supplementary Figure S2: Representative raw UV images of RT-PCR gels for two samples: one from cord blood (CB) and one from bone marrow (BM). The left panel shows uncropped, full-length gel images from CB samples, including Lin-sorted (CB), $CD34^{+}$ (CB-sorted), and $CD34^{-}$ (CB-sorted) samples. The right panel presents uncropped, full-length gel images from BM samples, including unsorted (BM), $CD34^{+}$ (BM-sorted), and $CD34^{-}$ (BM-sorted) samples.

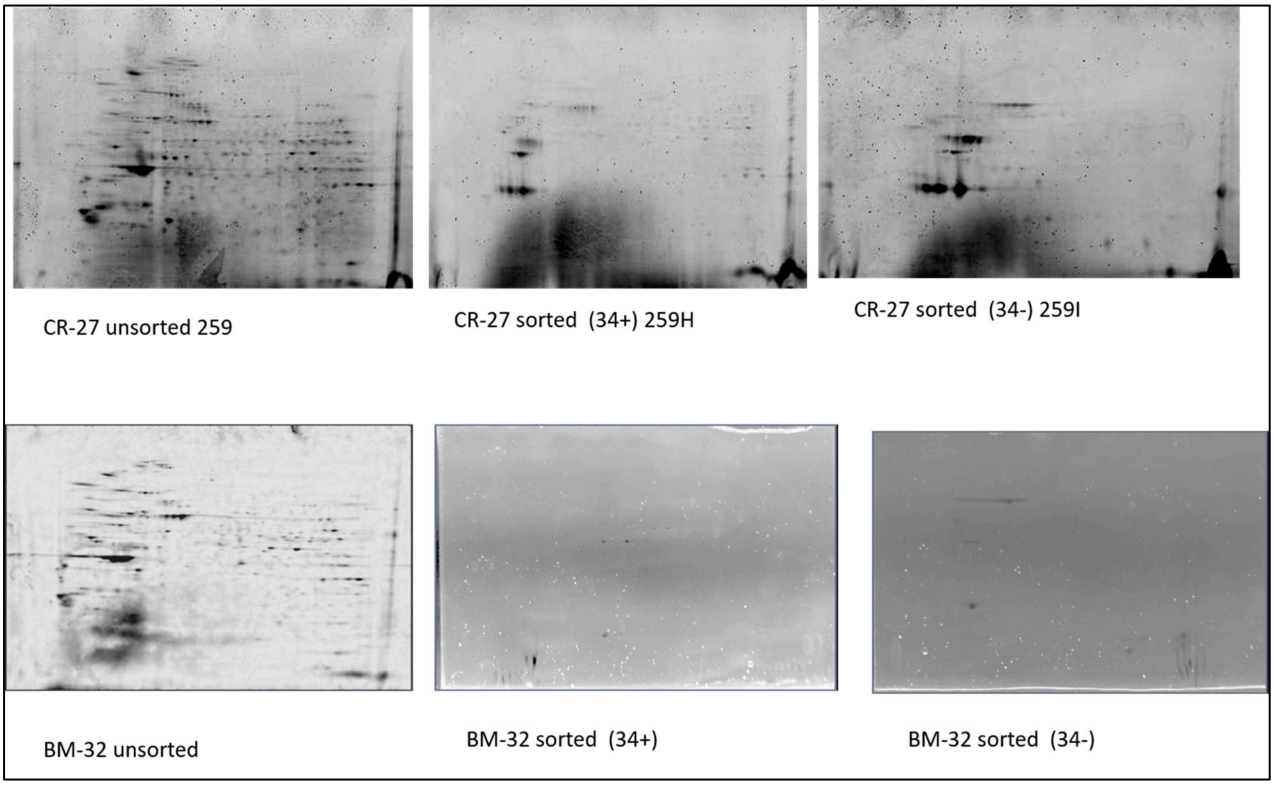

Supplementary Figure S3--Representative 2-DE(Upper panel) Un cropped entire images from unsorted (CR-27 Unsorted), CD34+ (CR-27 sorted) and CD34− (CR-27sorted) cord blood samples. (Lower Panel) Un dropped entire images from unsorted (BM-32), CD34+ (BM-32 sorted), and CD34− (BM-32 sorted) Bone Marrow samples.

Figure S4A

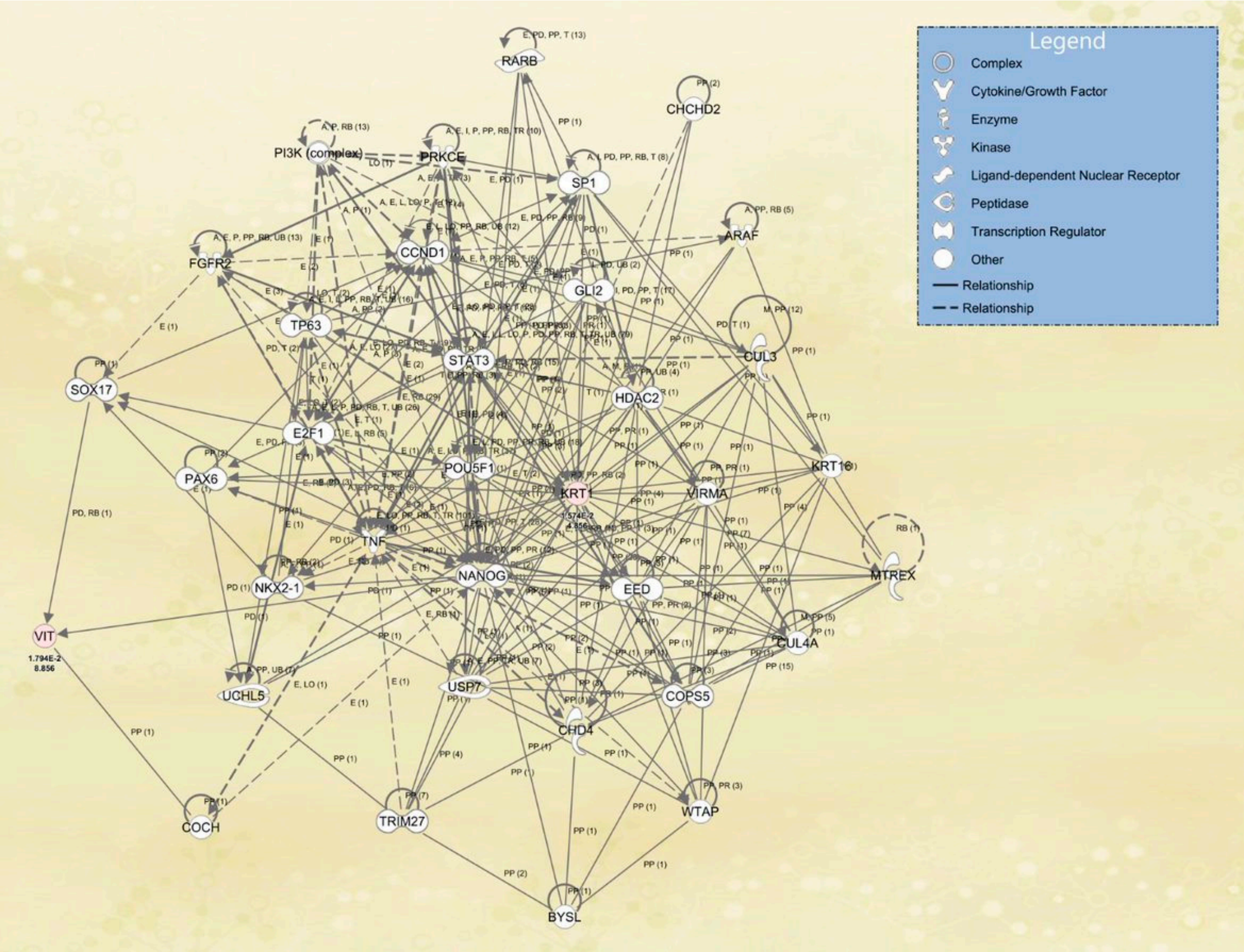
Legend
Complex
Cytokine/Growth Factor
Enzyme
Kinase
Ligand-dependent Nuclear Receptor
Peptidase
Transcription Regulator
Other
Relationship
Relationship
RARB
CHCHD2
PI3K (complex)
PRKCE
SP1
ARAF
FGFR2
CCND1
GLI2
TP63
STAT3
CUL3
SOX17
HDAC2
E2F1
PAX6
POU5F1
KRT1
VIRMA
KRT18
TNF
NANOG
EED
MTREX
NKX2-1
VIT
1.794E-2
8.856
CUL4A
UCHL5
USP7
COPS5
CHD4
COCH
TRIM27
WTAP
BYSL

Figure S4B

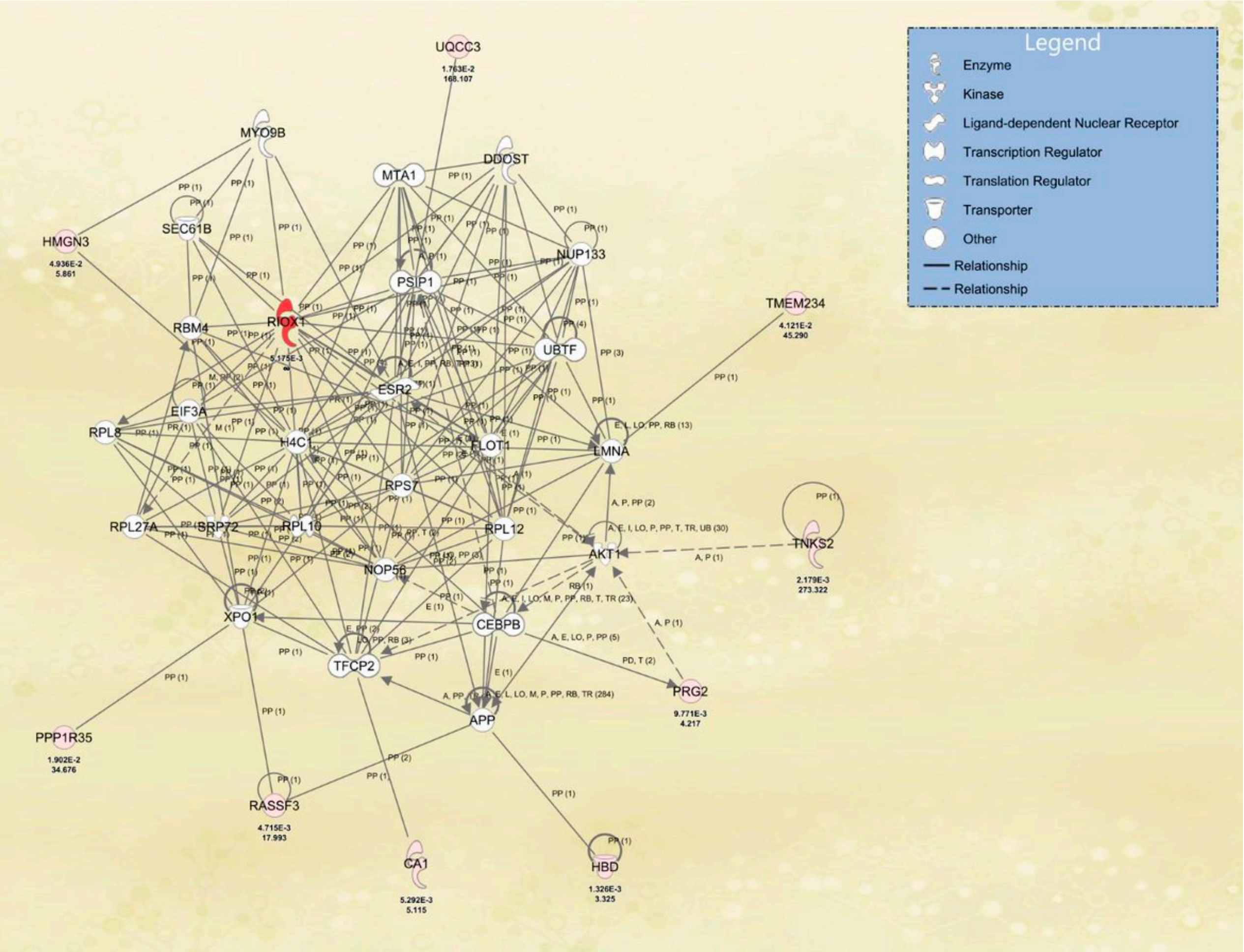

Figure S4A. Representation of the Ingenuity Pathway Analysis (IPA) of some of the 44 differentially expressed proteins (+/–) and (–/–) HSC/HPC subsets. (a) Highlighted in pink are some of the identified proteins involved in gene expression, protein synthesis, and RNA damage and repair signaling pathways. Grey represents the other molecules in the IPA database that made up the entire network. (B) Other molecules were implicated in cellular development, and embryonic development signaling pathways. In the core of the network is Nanog and stat3 with other interacting proteins. The network analysis was generated using IPA (https://www.qiagenbioinformatics.com). **Table S1. List of 44 differentially expressed proteins in CD34+ *versus* CD34¯ HSC/HPCs cells**

Figure S5A

Lin^{-}CD34^{+}CD38^{-}

Lin^{-}CD34^{-}CD38^{-}

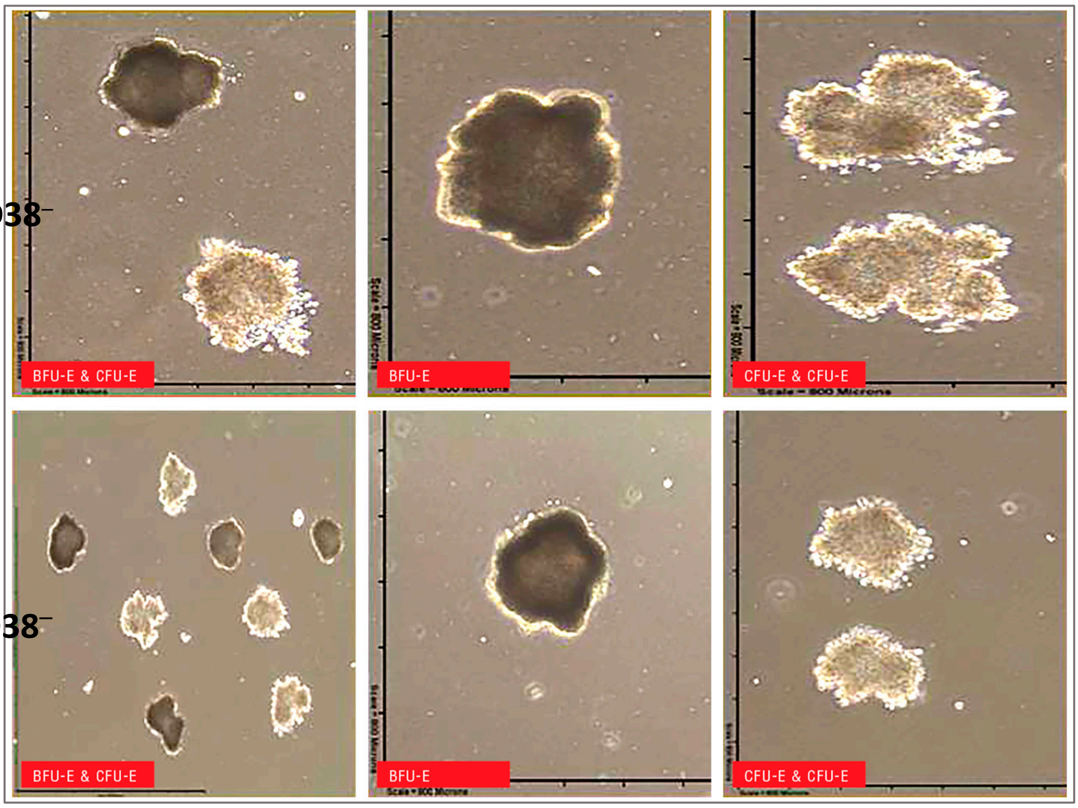

Figure S5B

Lin^{-}CD34^{+}CD38^{-}

Lin^{-}CD34^{+}CD38^{-}

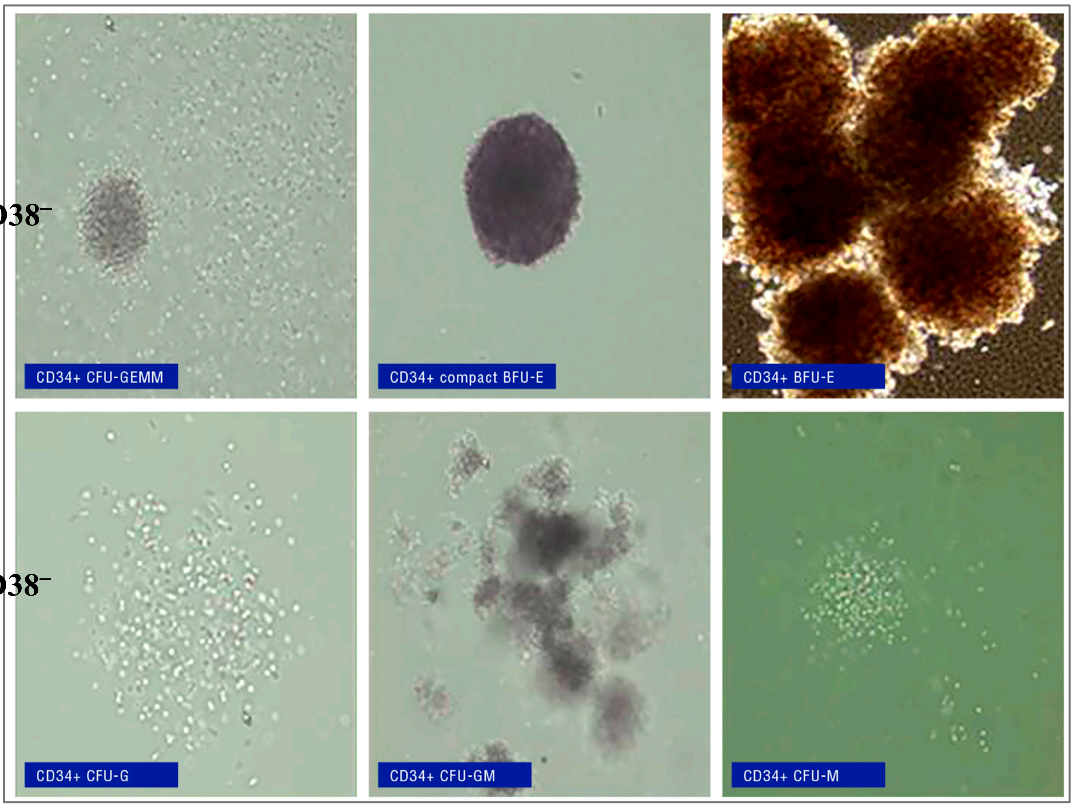

Figure S5. Displays representative results of Colony-forming assays from eight CB samples. (A) Both (+/−) and (−/−) HSC/HPC subsets were sorted from 8 CB samples and cultured for 14 days. Colony formation - erythroid progenitor colonies namely burst-forming unit-erythroid (BFU-E) and colony-forming unit-erythroid (CFU-E) were measured in both CD34+and $CD34^{-}$subsets. (B) Whereas, CFU-E, BFU-E, CFU-G, CFU-M, CFU-GM, and CFU-GEMM colonies were only produced by CD34+ fraction.

Table S1: Presents the absolute numbers of CBMCs and the Lin-CD34+CD38 Low/- HSC/HPC to Lin-CD38 Low/- HSC/HPC fractions in 40 CB samples.

Sample ID	*Number of CBMCs cells before sorting X 10^6*	*$Lin^{-}CD34+CD38^{-}$ X 10^3*	*$Lin^{-}CD34^{-}$ $CD38^{-}$ X 10^3*	*Sample Volume*	*PBMC /ml*
1	234.6	200	1000	125	1.88
2	234.6	200	1000	125	1.88
3	108.7	50	120	135	0.81
4	59.4	926	62	140	0.42
5	74.33	91	120	115	0.65
6	28.13	30	75	110	0.26
7	100.3	30	133	125	0.80
8	15.6	38.399	82.683	68	0.23
9	415	151	1100	125	3.32
10	185	240	1500	175	1.06
11	33.7	62	480	150	0.22
12	22.25	7	60	75	0.30
13	3.4	13	229	90	0.04
14	170.45	20	160	125	1.36
15	69.2	21	280	115	0.60
16	74.3	8	166	110	0.68
17	80.4	10	50	75	1.07
18	41.8	37	19	110	0.38
19	34.6	11	56	90	0.38
20	24.9	370	1400	135	0.18
21	9.1	370	560	125	0.07
22	11.9	140	400	70	0.17
23	25	555	1600	99	0.25
24	55	1.03	1550	125	0.44
25	12	21	167	76	0.16

26	17.8	30	153	69	0.26
27	9	76	333	55	0.16
28	34	140	270	60	0.57
29	10.2	45	71	60	0.17
30	13	74	130	80	0.16
31	18.3	154	598	100	0.18
32	30.1	15	870	124	0.24
33	45	208	1.924	150	0.30
34	51	72	425	100	0.51
35	25	52	270	63	0.40
36	37.5	343	325	125	0.30
37	75	45	296	55	1.36
38	65	614	1030	100	0.65
39	25	263	281	90	0.28
40	17	85	119	92	0.18
41	21	297	182	120	0.18

Table S2. List of 44 differentially expressed proteins in CD34+ *versus* CD34¯ HSC/HPCs cells

Accession	Peptide count	Unique peptides	Anova (p)	Max fold change	Highest mean condition	Lowest mean condition	Description
P10412	10	4	0.032	2.07	CD34+	CD34−	Histone H1.4 OS=Homo sapiens GN=HIST1H1E
Q96KK5	8	6	0.005	2.34	CD34+	CD34−	Histone H2A type 1-H OS=Homo sapiens GN=HIST1H2AH
P52907	2	1	0.003	2.44	CD34+	CD34−	F-actin-capping protein subunit alpha-1 OS=Homo sapiens GN=CAPZA1
P36957	1	1	0.007	2.46	CD34+	CD34−	Dihydrolipoyllysine-residue succinyltransferase component of 2-l OS=Homo sapiens GN=DLST
P09429	7	3	0.010	2.55	CD34+	CD34−	High mobility group protein B1 OS=Homo sapiens GN=HMGB1
P08238	16	8	0.028	2.57	CD34−	CD34+	Heat shock protein HSP 90-beta OS=Homo sapiens GN=HSP90AB1
P16401	9	6	0.012	2.59	CD34+	CD34−	Histone H1.5 OS=Homo sapiens GN=HIST1H1B
Q75002	1	1	0.012	2.62	CD34−	CD34+	Gag-Pol polyprotein OS=Human immunodeficiency virus type 1 group M subtype C (isolate ETH2220) GN=gag-pol

P61978	9	9	0.003	2.73	CD34+	CD34−	Heterogeneous nuclear ribonucleoprotein K OS=Homo sapiens GN=HNRNPK
P08670	30	29	0.046	2.73	CD34−	CD34+	Vimentin OS=Homo sapiens GN=VIM
P22492	5	2	0.003	2.79	CD34+	CD34−	Histone H1t OS=Homo sapiens GN=HIST1H1T
P68871	12	8	0.001	2.98	CD34+	CD34−	Hemoglobin subunit beta OS=Homo sapiens GN=HBB
O60506	6	2	0.020	3.04	CD34+	CD34−	Heterogeneous nuclear ribonucleoprotein Q OS=Homo sapiens GN=SYNCRIP
Q8IUE6	3	1	0.010	3.05	CD34+	CD34−	Histone H2A type 2-B OS=Homo sapiens GN=HIST2H2AB
P51149	3	2	0.035	3.12	CD34+	CD34−	Ras-related protein Rab-7a OS=Homo sapiens GN=RAB7A
O75367	11	10	0.020	3.14	CD34+	CD34−	Core histone macro-H2A.1 OS=Homo sapiens GN=H2AFY
P37837	8	8	0.015	3.15	CD34+	CD34−	Transaldolase OS=Homo sapiens GN=TALDO1
P62805	11	11	0.001	3.15	CD34+	CD34−	Histone H4 OS=Homo sapiens GN=HIST1H4A
P02042	5	1	0.001	3.32	CD34+	CD34−	Hemoglobin subunit delta OS=Homo sapiens GN=HBD
P11021	13	12	0.020	3.37	CD34−	CD34+	78 kDa glucose-regulated protein OS=Homo sapiens GN=HSPA5
P62888	1	1	0.006	3.38	CD34+	CD34−	60S ribosomal protein L30 OS=Homo sapiens GN=RPL30
P54652	9	1	0.008	3.40	CD34+	CD34−	Heat shock-related 70 kDa protein 2 OS=Homo sapiens GN=HSPA2
P07205	4	1	0.031	3.59	CD34+	CD34−	Phosphoglycerate kinase 2 OS=Homo sapiens GN=PGK2
P00558	13	10	0.031	3.59	CD34+	CD34−	Phosphoglycerate kinase 1 OS=Homo sapiens GN=PGK1
P06733	23	22	0.004	3.69	CD34+	CD34−	Alpha-enolase OS=Homo sapiens GN=ENO1
P13727	9	9	0.010	4.22	CD34+	CD34−	Bone marrow proteoglycan OS=Homo sapiens GN=PRG2
P04264	4	3	0.016	4.86	CD34+	CD34−	Keratin_ type II cytoskeletal 1 OS=Homo sapiens GN=KRT1
P00915	3	2	0.005	5.12	CD34−	CD34+	Carbonic anhydrase 1 OS=Homo sapiens GN=CA1
Q15651	2	2	0.049	5.86	CD34−	CD34+	High mobility group nucleosome-binding domain-containing protein 3 OS=Homo sapiens GN=HMGN3
P40939	1	1	0.004	6.88	CD34+	CD34−	Trifunctional enzyme subunit alpha_ mitochondrial OS=Homo sapiens GN=HADHA

P04259	7	6	0.031	6.88	CD34+	CD34−	Keratin_ type II cytoskeletal 6B OS=Homo sapiens GN=KRT6B
P32119	9	8	0.029	8.29	CD34−	CD34+	Peroxiredoxin-2 OS=Homo sapiens GN=PRDX2
Q6UXI7	3	3	0.018	8.86	CD34−	CD34+	Vitrin OS=Homo sapiens GN=VIT
Q6ZRM9	1	1	0.031	8.87	CD34−	CD34+	Putative uncharacterized protein FLJ46235 OS=Homo sapiens
P48735	4	4	0.002	9.25	CD34+	CD34−	Isocitrate dehydrogenase [NADP]_ mitochondrial OS=Homo sapiens GN=IDH2
Q13360	2	2	0.016	10.87	CD34−	CD34+	Zinc finger protein 177 OS=Homo sapiens GN=ZNF177
Q86WH2	1	1	0.005	17.99	CD34−	CD34+	Ras association domain-containing protein 3. OS=Homo sapiens GN=RASSF3
P01903	1	1	0.002	25.57	CD34+	CD34−	HLA class II histocompatibility antigen_ DR alpha chain OS=Homo sapiens GN=HLA-DRA
Q8TAP8	1	1	0.019	34.68	CD34−	CD34+	Protein phosphatase 1 regulatory subunit 35 OS=Homo sapiens GN=PPP1R35
Q8WY98	1	1	0.041	45.29	CD34−	CD34+	Transmembrane protein 234 OS=Homo sapiens GN=TMEM234
P03450	12	1	0.021	68.78	CD34−	CD34+	Tubulin beta-4A chain OS=Homo sapiens GN=TUBB4A
Q6UW78	1	1	0.018	168.11	CD34−	CD34+	Ubiquinol-cytochrome-c reductase complex assembly factor 3 OS=Homo sapiens GN=UQCC3
Q9H2K2	1	1	0.002	273.32	CD34−	CD34+	Tankyrase-2 OS=Homo sapiens GN=TNKS2
Q9H6W3	3	2	0.005	Infinity	CD34+	CD34−	Ribosomal oxygenase 1 OS=Homo sapiens GN=RIOX1
